# Supplementary material for: Structural and Functional Analysis of Disease-Linked p97 ATPase Mutant Complexes
Source: Int J Mol Sci. 2021 Jul 28;22(15):8079. doi: 10.3390/ijms22158079 (PMC8347982; doi:10.3390/ijms22158079)
Supplement: Supplementary file 1 [file ijms-22-08079-s001.zip › ijms-1304320-supplementary.pdf]

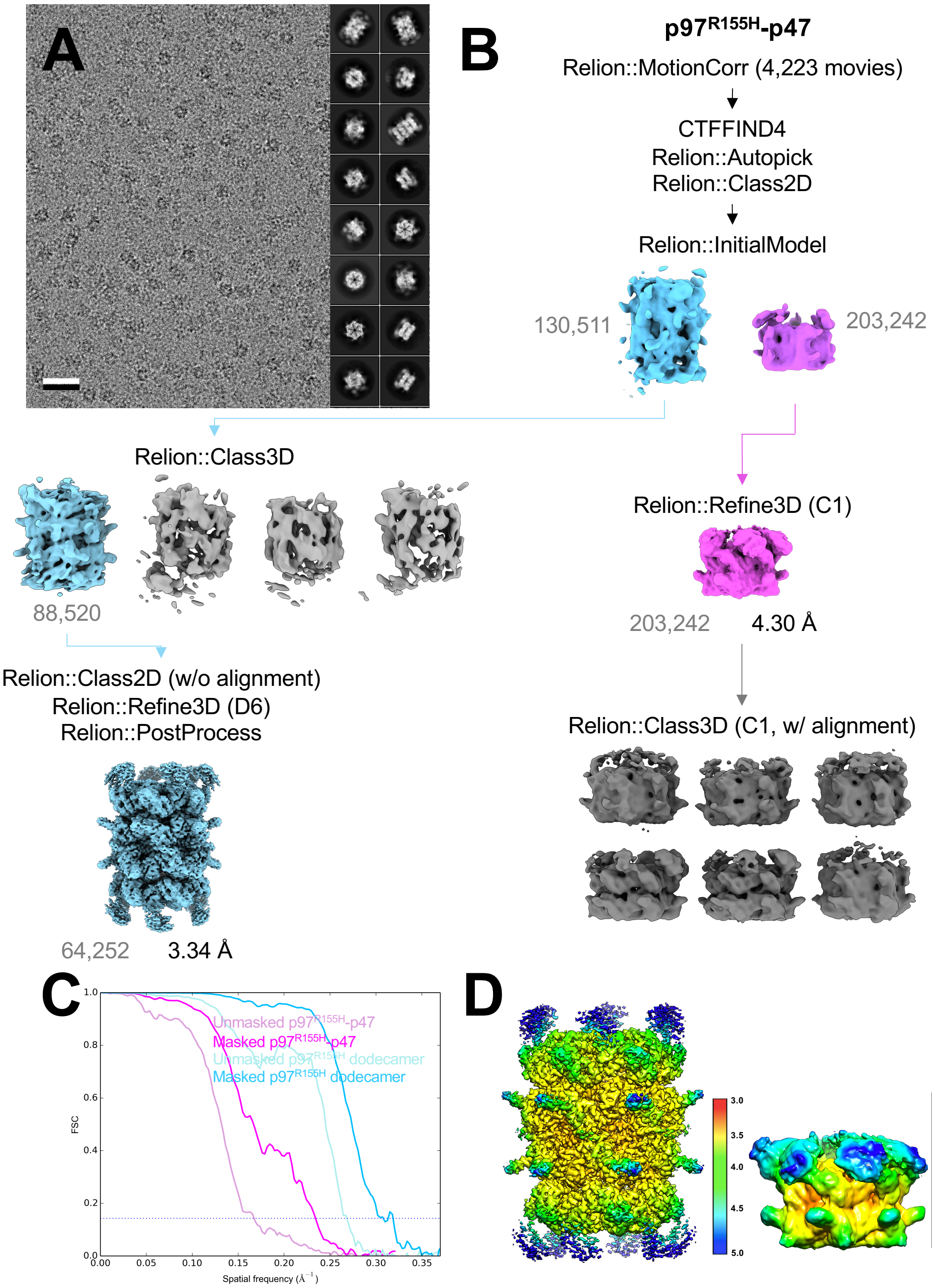

Figure S1. Cryo-EM analysis of the full-length p97^{R155H}-p47 assembly in the absence of nucleotides.

(A) Electron micrograph of cryogenic p97^{R155H}-p47 complexes. Black contrast represents protein, and white is background. Scale bar indicates 50 nm. The inset to the side shows representative 2D class averages. Box side length is 374 Å.

(B) Image processing flowchart for single-particle cryo-EM reconstruction.

(C) Fourier-shell correlation (FSC) plot of the 3D reconstructions.

(D) Local resolution estimation of the reconstructions.

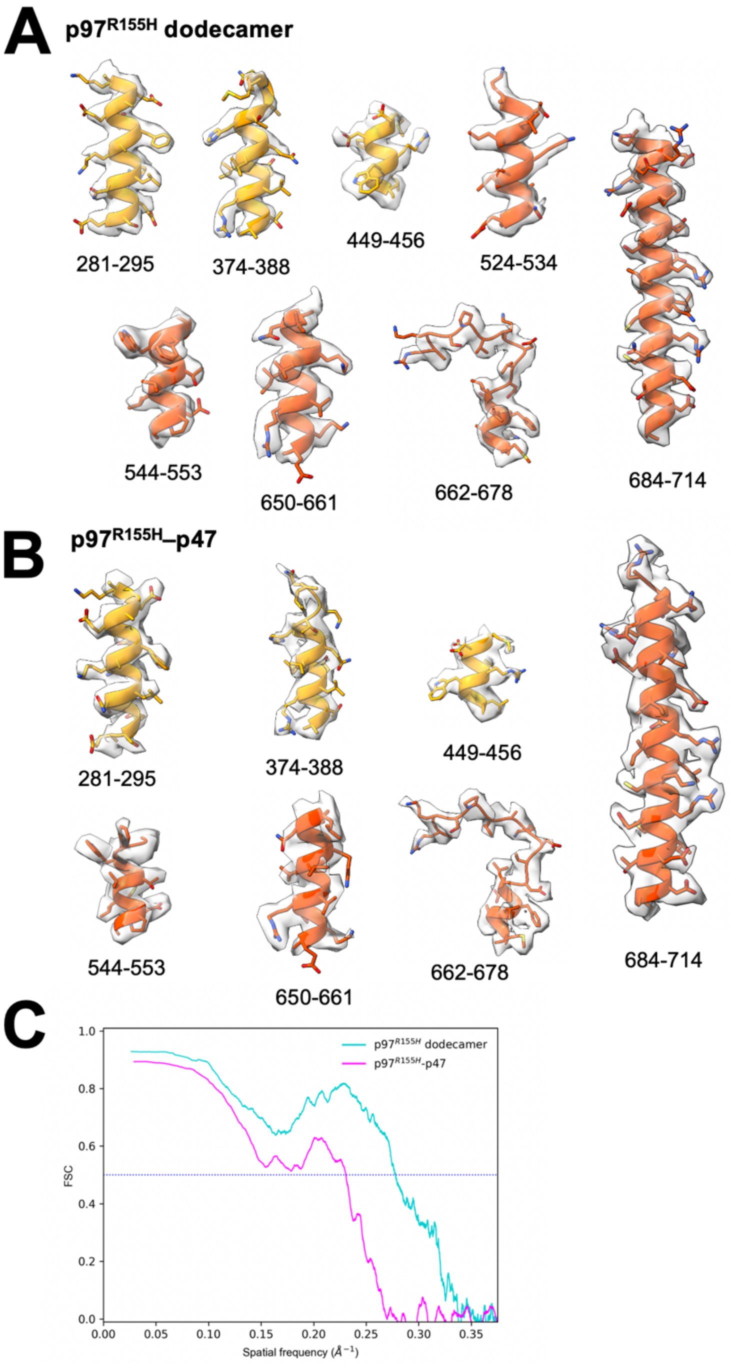

Figure S2. Modelling of atomic coordinates in the cryo-EM densities of the p97^{R155H} dodecamer and p97^{R155H}-p47 complexes in the absence of nucleotide.

(A) Model fitting of the p97^{R155H} dodecamer. Orange and orange red are D1 and D2 domains, respectively.

(B) Model fitting of the p97^{R155H}-p47 assembly.

(C) Fourier-shell correlation (FSC) plot. Light blue and pink curves are p97^{R155H} dodecamer and p97^{R155H}-p47 assembly. Blue dash indicates where FSC values are 0.5.

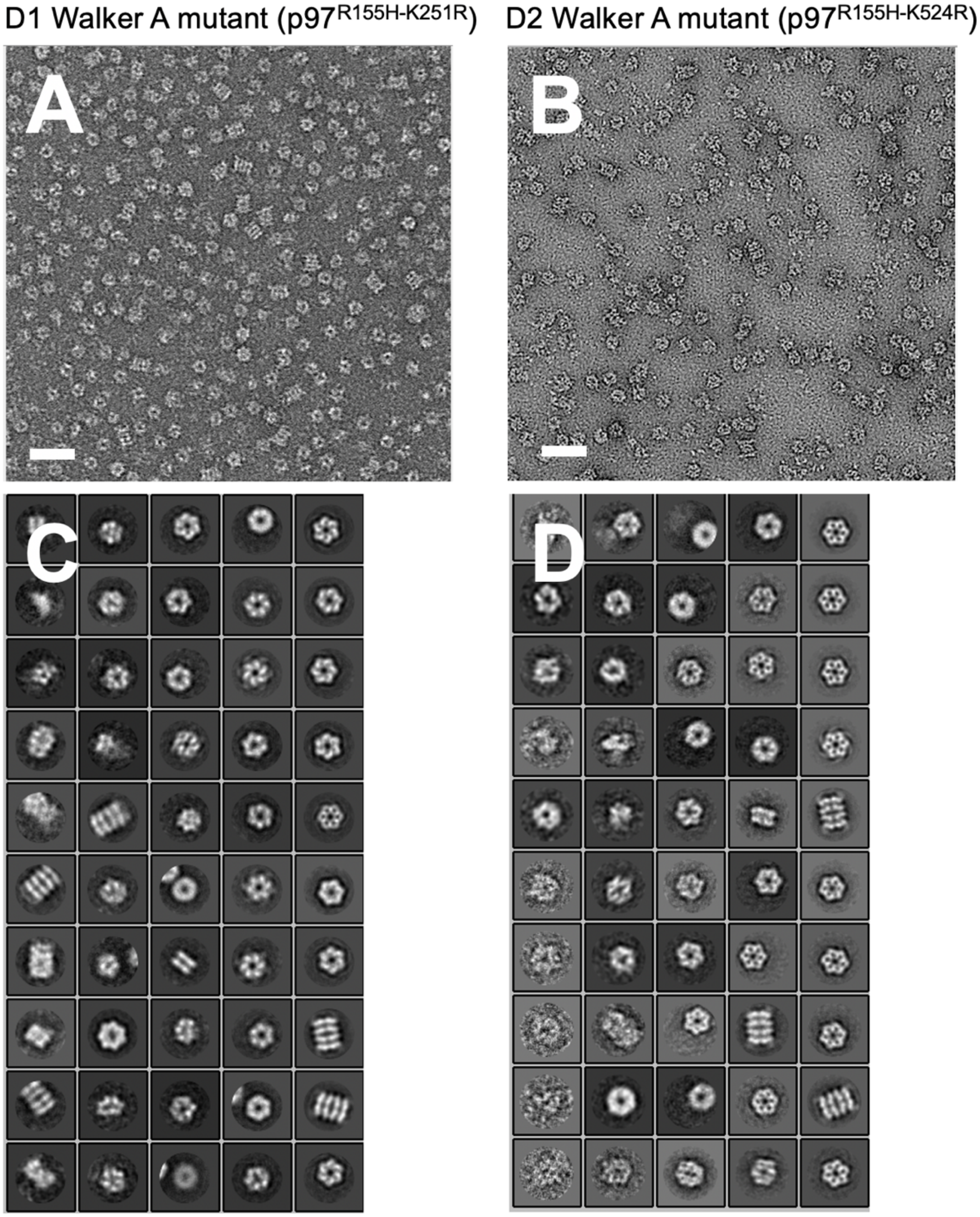

Figure S3. Single-particle EM image analysis on the negatively stained Walker A mutants in the context of p97^{R155H}.

Electron images of the negatively stained (A) p97$^{R155H\text{-}K251R}$ and (B) p97$^{R155H\text{-}K524R}$ double mutants are shown. Scale bars indicate 50 nm. Two-dimensional class averages of selected particle images were calculated in (C) and (D), respectively. Box side lengths are 333 Å.

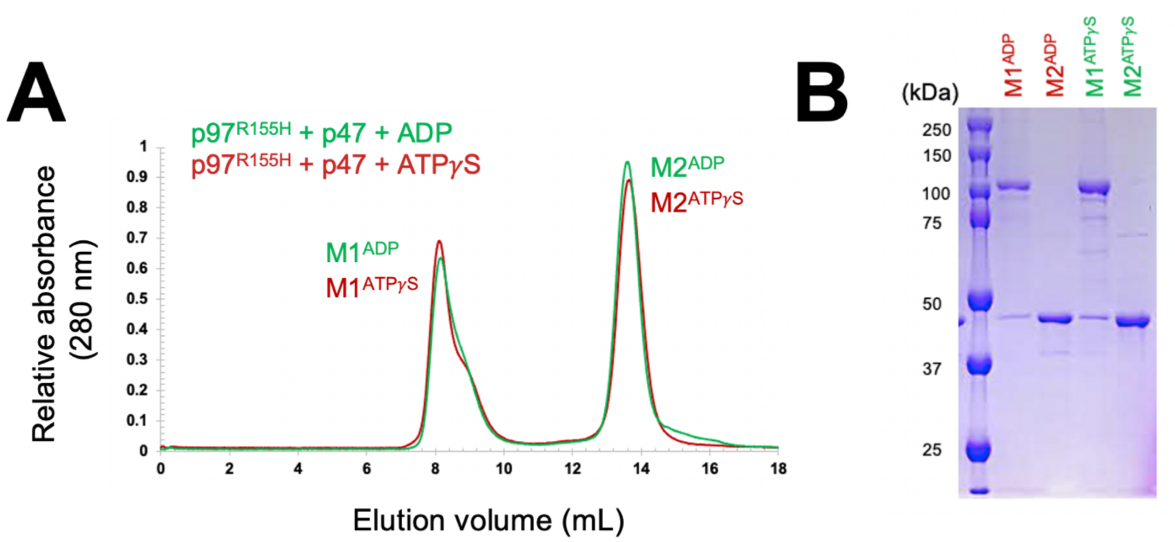

Figure S4. Biochemical characterization of the p97$^{R155H}|_{ADP}$-p47 and p97$^{R155H}|_{ATP\gamma S}$-p47.

(A) Size-exclusion chromatographic (SEC) profiles. Green and red curves represent p97$^{R155H}|_{ADP}$ and p97$^{R155H}|_{ATP\gamma S}$-p47, respectively.

(B) SDS-PAGE analysis of the SEC eluted peaks of the complex assemblies.

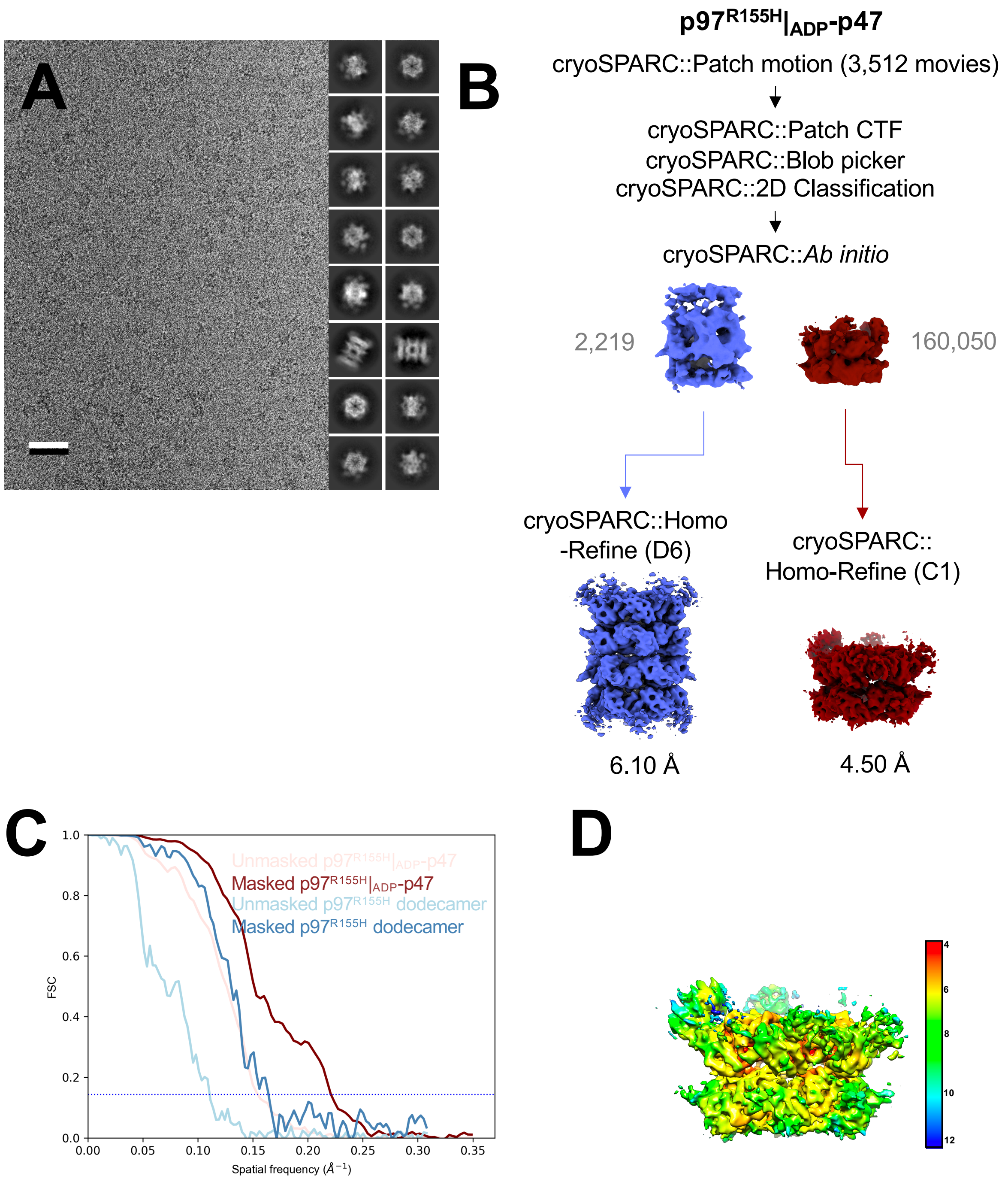

Figure S5. Cryo-EM structural analysis of the full-length p97^{R155H}|$_{ADP}$-p47 assembly.
(A) Electron micrograph of cryogenic preparation of the p97^{R155H}|$_{ADP}$-p47 complex. Black contrast represents protein and white is background. Scale bar indicates 50 nm. Insets to the side show representative 2D class averages. Box side length is 382 Å.
(B) Image processing flowchart for single-particle cryo-EM reconstruction.
(C) Fourier-shell correlation (FSC) plot of the 3D reconstructions.
(D) Local resolution estimation of the reconstructions.

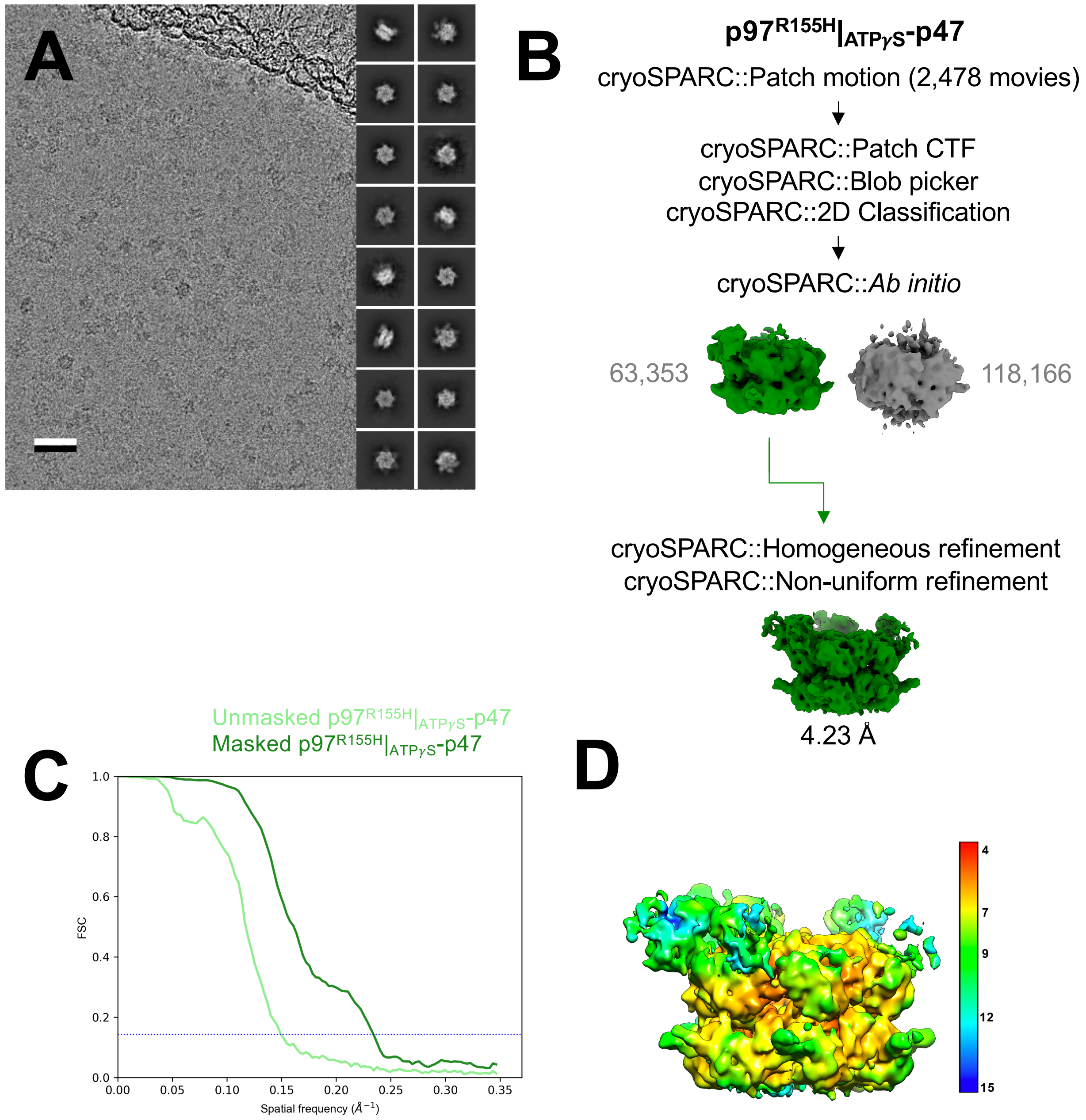

Figure S6. Cryo-EM structural analysis of the full-length $p97^{R155H}|_{ATP\gamma S}$-p47 assembly.
(A) Electron micrograph of the cryogenic $p97^{R155H}|_{ATP\gamma S}$-p47 complex. Black contrast represents proteins and white is background. Scale bar indicates 50 nm. Inset shows representative 2D class averages. Box side length is 382 Å.
(B) Image processing flowchart for single-particle cryo-EM reconstruction.
(C) Fourier-shell correlation (FSC) plot of the 3D reconstructions.
(D) Local resolution estimation of the reconstructions.

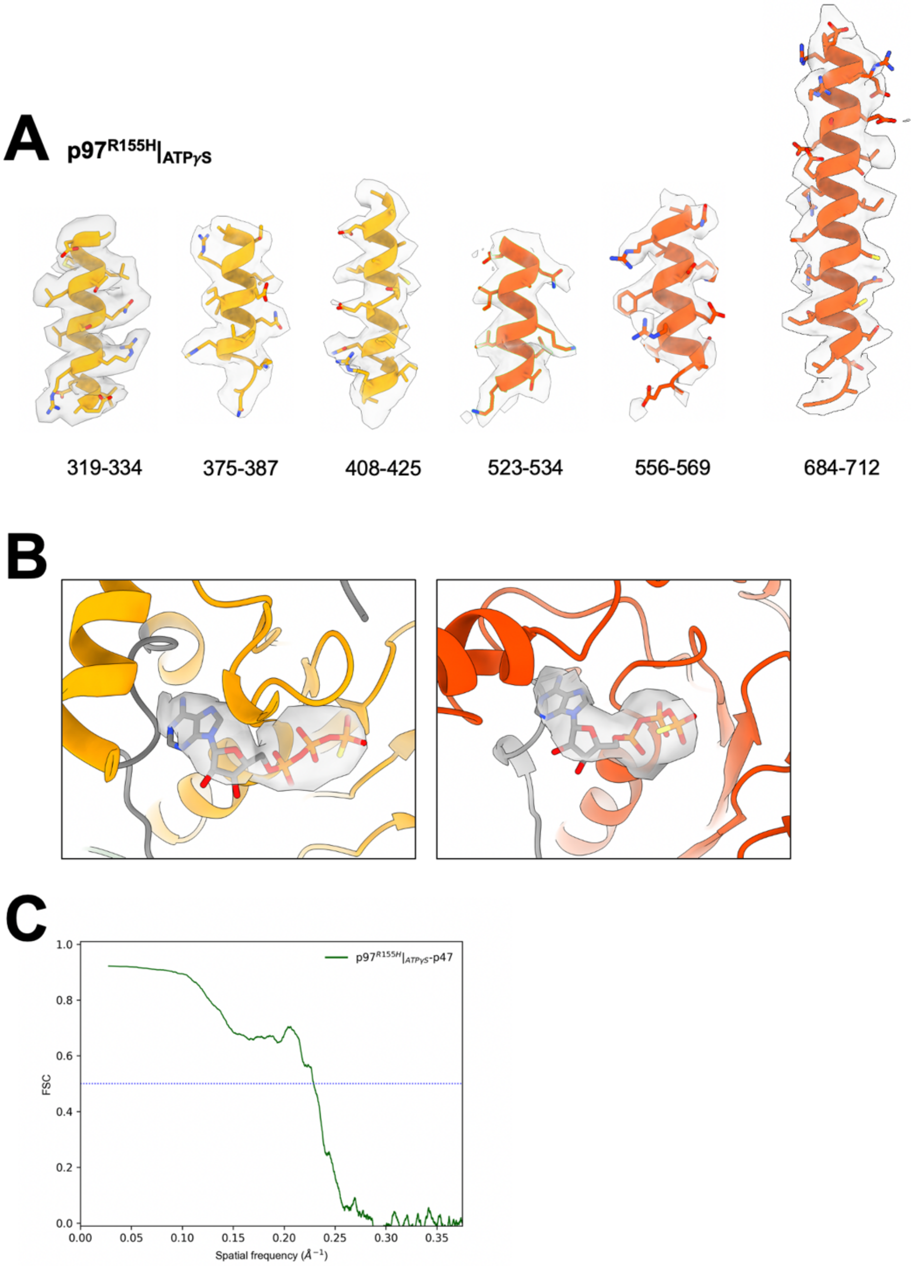

Figure S7. Modelling of atomic coordinates into the cryo-EM densities of p97^{R155H}|$_{ATP\gamma S}$-p47 complexes.

(A) Model fitting of the p97^{R155H}|$_{ATP\gamma S}$-p47 assembly. Orange and orange red are D1 and D2 domains, respectively.

(B) Model fitting of ATPγS nucleotides in D1 (left) and D2 (right) binding sites.

(C) Fourier-shell correlation (FSC) plot. Green curve is p97^{R155H}|$_{ATP\gamma S}$-p47 assembly. Blue dash indicates FSC values of 0.5.

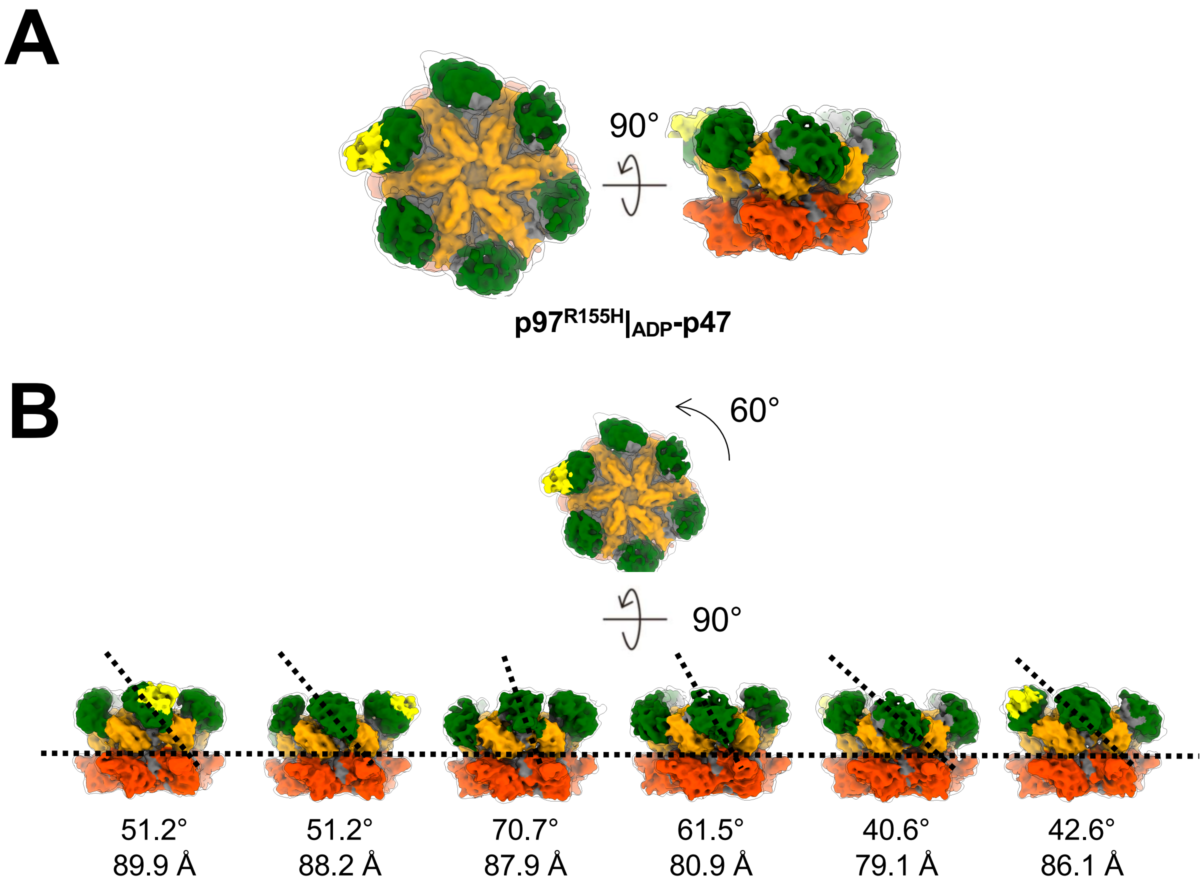

Figure S8. Cryo-EM structure of the p97^{R155H}|$_{ADP}$-p47 complex.

(A) Cryo-EM densities of p97^{R155H}|$_{ADP}$-p47. Green, orange, orange red and yellow indicate the NTD, D1 and D2 domains of the p97^{R155H} and p47^{UBX}, respectively. Grey indicates the densities for the linkers between domains of p97^{R155H}. Envelopes are the cryo-EM maps at a lower contour (1.0σ).

(B) NTD conformations. The tilting angles of the NTDs are labeled, and the measured heights are the distance between the NTD center and the bottom of the D2 ring plane.

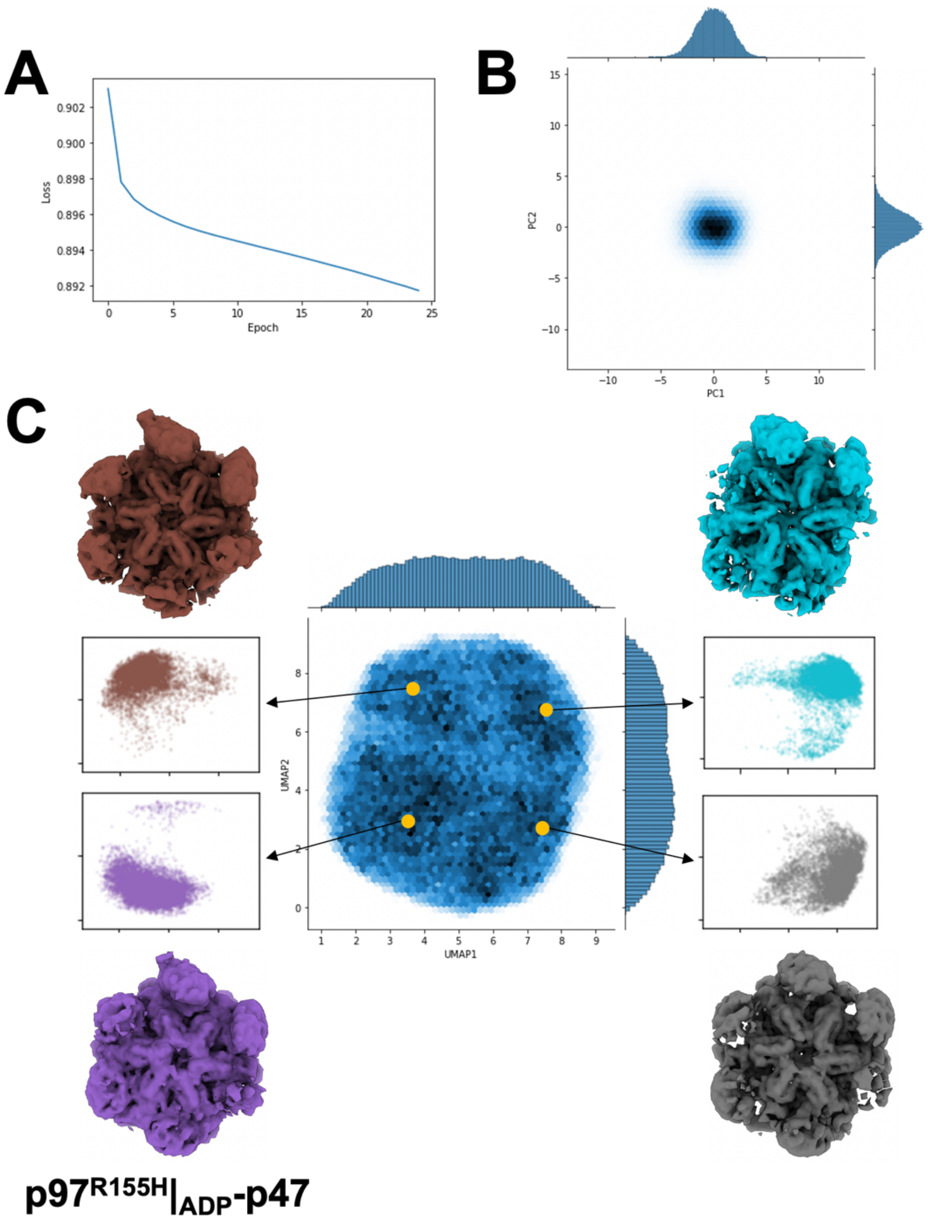

Figure S9. Deep coordinate neural network analysis of the p97^{R155H}|$_{ADP}$-p47 single-particle cryo-EM images.

(A) Plot of the loss function versus learning epochs. The monotonic decrease of the losses in the later epochs shows the convergence of the model weights.

(B) Plot of the two major principal components in the hyperspace. The horizontal axis is the first principal axis, and the vertical axis is the second principal axis. The blue density presents the particle image numbers. Because the distribution of the particle images is isotropic, it is not possible to differentiate the heterogeneity effectively using the principal component analysis (PCA) plot.

(C) UMAP representation and the averaged reconstructions of the four selected clusters. The four reconstructions show the invariable D1 and D2 rings and the heterogeneous NTD densities.

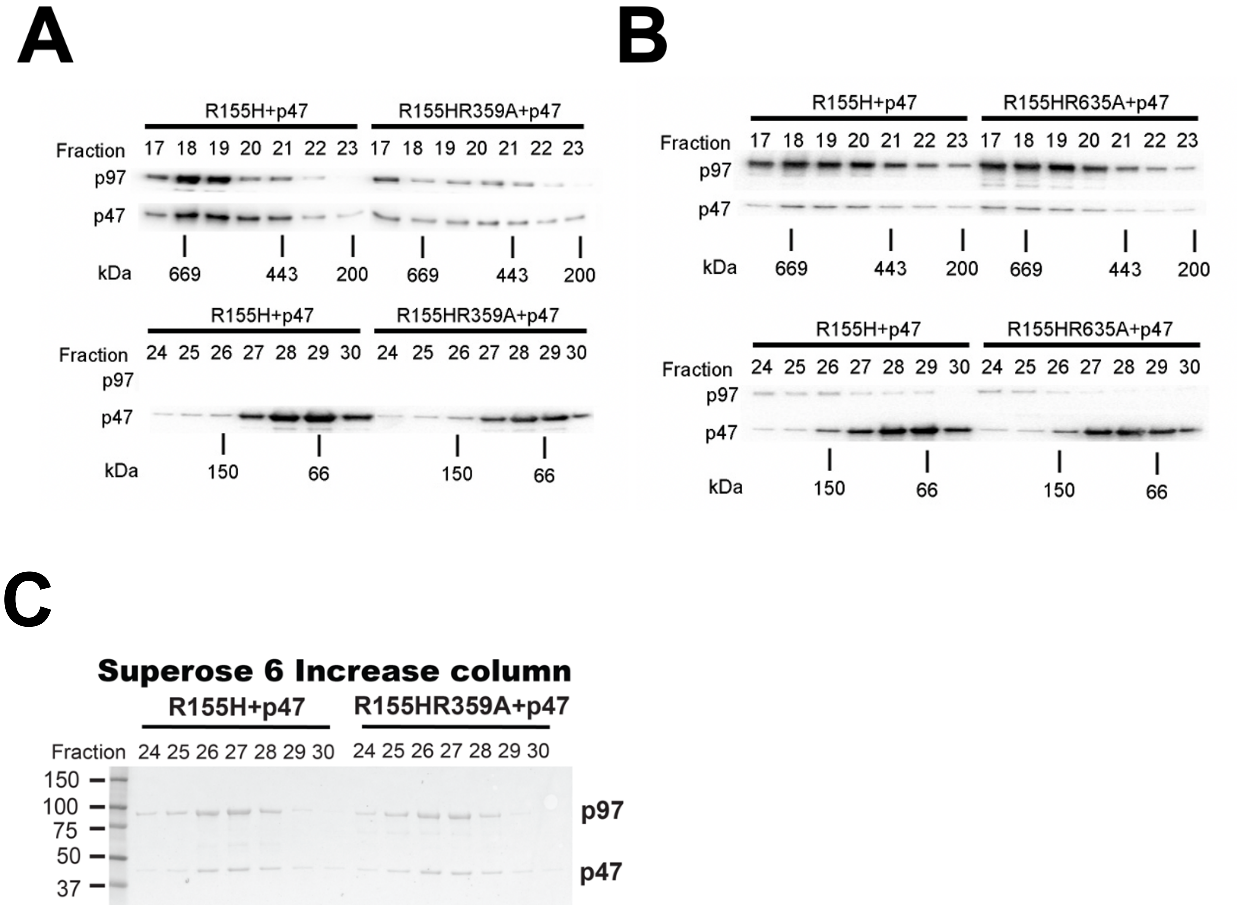

Figure S10. Western blotting analyses of the SEC-eluted fractions of the p97-p47 complexes.

The SEC of p97-p47 complex was performed using a Superdex 20010/300 GL column and subsequent Western blotting was performed for the SEC fractions from (A) 17 to 23 and (B) 24 to 30.

(C) The SEC of p97-p47 complex was performed using a Superose 6 Increase column that can separate the molecules with molecular weights between 5 kDa and 5 MDa. The bands were detected using Ponceau S staining.

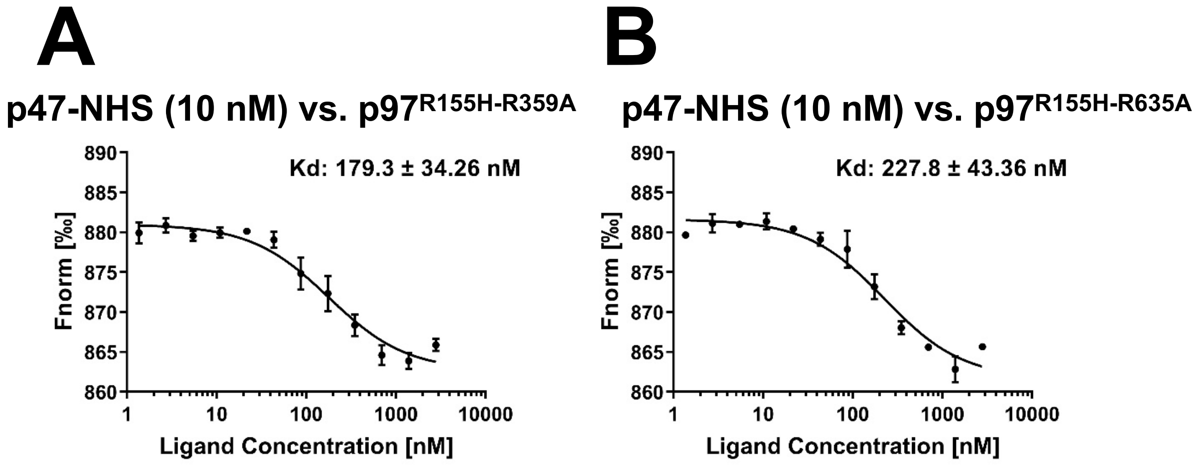

Figure S11. Determination of the binding constant, K_d, for the interaction between p97 arginine finger mutants and p47 proteins.

Temperature-related intensity change (TRIC) signals on a DianthusNT.23 PicoDuo instrument (Nano-Temper Technologies) was used to determine the equilibrium binding constant (K_d) for p47 in solution with four p97 proteins: (A) p97$^{R155H\text{-}R359A}$ and (B) p97$^{R155H\text{-}R635A}$. Unlabeled p97 protein was titrated against 10 nM of RED-NHS-labeled p47 in two-fold steps from 2,800 nM to 1.37 nM.

Table S1. List of plasmids used for overexpression of p97 and p47 proteins.

Plasmid Number	Plasmid name	Vector	Source and Reference
TCB-328	Rat p47 pET15_T	pET15b_TEV linker	Chou et al. 2014
TCB-197	Human p97 pET15_T	pET15b_TEV linker	Chou et al. 2014
TCB-210	Human R155H p97 pET15_T	pET15b_TEV linker	Chou et al. 2014
TCB-423	Human R155H, K251R p97 pET15_T	pET15b_TEV linker	This study
TCB-436	Human R155H, K524R p97 pET15_T	pET15b_TEV linker	This study
TCB-460	Human R155H, R359A p97pET15T_T	pET15b_TEV linker	This study
TCB-461	Human R155H, R635A p97pET15T_T	pET15b_TEV linker	This study

Chou TF, Bulfer SL, Weihl CC, Li K, Lis LG, Walters MA, Schoenen FJ, Lin HJ, Deshaies RJ, Arkin MR (2014) Specific inhibition of p97/VCP ATPase and kinetic analysis demonstrate interaction between D1 and D2 ATPase domains. *J Mol Biol* 426:2886-99

Table S2. Statistics of single-particle cryo-EM structure determination on p97-p47 assembly.

**Protein**		p97^{R155H} – p47			
Data collection					
**Electron microscope**			Thermo Fisher/FEI Titan Krios TEM		
**Accelerating voltage (kV)**			300		
**Spherical aberration constant (mm)**			2.7		
**Detector camera**			Gatan K2 Summit DED camera		
**Defocus (μm)**			-0.8 - -2.5		
**Nominal magnification**			48,077×		
**Physical pixel size (Å/pixel)**			1.04		
**Image dose (e^-/Å^2)**			44.3		
Image processing					
**Number of movies**		4,223		3,512	2,796
**Number of particles selected (initial)**		368,575		1,124,232	401,303
**Group**	p97^{R155H} dodecamer (no nucleotides) EMD-23191 PDB code: 7L5W	p97^{R155H}-p47 (no nucleotides) EMD-24305 PDB code: 7R7U	p97^{R155H} dodecamer EMD-24306	p97^{R155H}-p47 (ADP) EMD-24304 PDB code: 7R7T	p97^{R155H}-p47 (ATPγS) EMD-24302 PDB code: 7R7S
**Number of particles used for final 3D density (final)**	64,252	203,242	2,219	160,050	63,353
**Spatial frequency at FSC of 0.143 (Å^{-1})**	3.34	4.30	6.10	4.50	4.23
**Imposed symmetry**	D6	C1	D6	C1	C1
**Sharpening *b*-factor (Å^2)**	-78.6	-139.3	-183.2	-122.3	-124.8
Modeling					
**Initial model used (PDB code)**	5FTK	5FTK		5FTK	5FTN 1S3S
**Model resolution (Å)**	3.5	4.8		4.4	4.3
**Model composition**					
**Non-hydrogen atoms**	51,828	25,362		35,351	33,239
**Protein residues**	6,636	3,240		4,482	4,219
**Ligands**					
**ADP**	0	0		12	0
**ATPγS**	0	0		0	12
***B* factors (Å^2)**					
**Protein**	43.9	128.6		281.4	117.1
**Ligands**	0	0		211.9	63.2
**RMS deviations**					
**Bond length (Å)**	0.006	0.004		0.004	0.004
**Bond angle (°)**	1.002	1.035		1.002	0.953
**Clash score**	11.15	7.09		6.34	6.53
**MolProbity score**	2.00	1.91		1.83	1.89
**Rotamer outlier (%)**	0.00	0.04		0.00	0.03

**Ramachandran plot (%)**					
**Disallowed**	0.00	0.00		0.00	0.10
**Allowed**	6.75	8.83		7.80	9.26
**Favored**	93.25	91.17		92.20	90.64
